# Adaptive calibration in a world of human universals

**DOI:** 10.3389/fpsyg.2026.1899620

**Published:** 2026-07-15

**Authors:** Catherine Salmon, Jessica Hehman

**Affiliations:** Psychology Department, University of Redlands, Redlands, CA, United States

**Keywords:** adaptive calibration, environmental impacts on development, human universals, individual differences, mismatch theory

## Introduction

A common complaint leveled historically at evolutionary approaches to human behavior is that they focus too much on human universals and not enough on human variation. However, over the last decade or so there has been greater recognition that this is an oversimplification of the adaptationist approach. The basis was in understanding human nature or what are referred to as human universals (Brown, 1991), such as sexual jealousy or incest avoidance. Such understanding provides solutions to human wide problems. Infidelity is a threat to high investment mating relationships. Over human history, males have faced the recurrent problem of paternity uncertainty and the specificity of male sexual jealousy can be viewed as an adaptive response designed to motivate behavior that will reduce the reproductive cost of sexual infidelity to men (Buss, 2018). Similarly, humans, as members of a social species, spend significant time with genetic relatives, including opposite sex relatives. However, selecting close kin as sexual partners is disadvantageous due to the risk of negatively impacting recessive mutations. While in some species, offspring disperse to avoid inbreeding, humans have done this much less frequently over our history. Thus, avoiding incest is an adaptive problem to solve, and indeed adaptations exist in the form of information processing systems that weigh cues of relatedness in order to avoid incest and its resulting costs (Lieberman and Patrick, 2023).

However, in addition to understanding human universals, there has also always been interest in understanding what shapes behavioral variation within and between individuals. Understanding what makes us human does not preclude understanding the variable ways in which our human nature is expressed. For example, not everyone experiences or expresses sexual jealousy the same way. Some individuals are more sensitive to cues of the likelihood of infidelity and this sensitivity can be influenced by within-couple mate value discrepancy, past history of experiencing infidelity, as well as other environmental factors such as the local sex ratio (Buss, 2018).

## Universal mechanisms and developmental calibration

A number of scholars have argued for the integration of evolutionary psychology and the field of individual differences (Buss, 2009; Kanazawa, 2011; Maestripieri and Boutwell, 2022). Heritable individual differences can be adaptive and functional. But in addition, differences in behavior can be contingent on current and past environmental calibration of universal psychological and physiological mechanisms (Del Giudice et al., 2011; Ellis and Del Giudice, 2019; Schwartz et al., 2023). Adaptationist approaches now and in the future should continue to examine the ways in which the universal architecture of human nature is calibrated to variable environmental conditions across the lifespan (Sell et al., 2017b).

Two examples of areas of research that have benefitted from evolutionary approaches that emphasize the role of the environment in calibrating psychological mechanisms are evolutionary developmental psychology's (evo-devo) approach to variation in parental investment and work on variations in female mate preferences.

### Parental investment

According to Trivers (1972), parental investment includes any parental effort that increases an offspring's chance of surviving and reproducing while reducing the parent's ability to invest in other current or future offspring. The adaptive problem associated with parental investment is how to allocate limited, zero-sum resources (time, energy, etc.) among current offspring and potential future offspring and maximize one's inclusive fitness. As with solutions to other universal human problems, evolutionary psychologists posit that adaptive allocation decisions are made automatically and on an unconscious level via universal neuroendocrine and psychological mechanisms designed by natural selection to attend to certain environmental cues and produce contingent responses based on those cues (Bugental, 2000; Daly and Wilson, 1980). The specific allocation of parental resources involves trade-offs as well as developmental consequences for both the offspring and the parent. As such, evolutionary psychologists have long been interested in identifying factors that influence individual differences in these parental investment decisions. What factors determine whether a parent invests less in more offspring or invests more in fewer offspring? Under what circumstances will parents invest heavily in current offspring, potentially risking their own survival and/or future reproductive opportunities, vs. conserve their resources for future offspring? What environmental cues influence how parents distribute their limited resources among multiple offspring (e.g., even or biased)? Daly and Wilson's (1984) investigation of infanticide cases provided some of the earliest evidence that parental investment decisions are influenced by the quality of the offspring (i.e., their reproductive potential), parental resource availability, and maternal age. Specifically, their investigation revealed that children with low reproductive potential, born to parents of low socioeconomic status, and/or born to younger mothers were at greater risk of neglect, maltreatment, and infanticide. Later work by Beaulieu and Bugental (2008) found evidence that, in addition to the previously observed main effects of offspring reproductive potential and parental resource availability, there was also an interaction between those two factors. When resources were high, mothers invested more in their low reproductive value infants (relative to their high reproductive children); and when resources were scarce, mothers invested less in their low reproductive value infants (relative to their high reproductive children). In more recent work, researchers proposed evo-devo models of how environmental factors generate variable adaptive responses leading to differences in parental behaviors (including parental investment) and their effects on child development both within and between families (Cabeza de Baca and Ellis, 2017). Using psychosocial acceleration theory, the researchers integrated evidence across different studies demonstrating that parental behaviors are contingent on many environmental cues in such a way to maximize inclusive fitness in that particular environment. Furthermore, their work suggests that exposure to environmental cues of instability and harshness in early childhood affects all domains of the child's further development, including physiological events (e.g., pubertal timing), mating behaviors, and stress reactivity.

### Female mate preferences

While lay people may misperceive the study of mate preferences to suggest that all females share the same preferences (and then claim that when research studies show otherwise, it is evidence against taking an adaptationist approach), the research is far more nuanced than that, particularly with regard to adaptive calibration of female mating strategies and preferences. For example, when considering the female perspective, a male's ability and willingness to protect their mate can be a double-edged sword in that the male aggressiveness and physical formidability that enables them to provide protection can also impose costs on female partners. As a result, females face a trade off that is likely to be influenced by the dangers of their current environment (or perhaps childhood environment depending on the plasticity of mate preference mechanisms) and individual differences in their perceived vulnerability to the threats of their environment. The benefits of such men should vary across individual women and environments. This may explain the mixed evidence on female preferences for masculinity in male faces and variation or lack thereof across the ovulatory cycle, though other research suggests that preferences for male upper body strength are more consistent (Sell et al., 2017a) and that women strongly value male willingness to provide protection (Barlev et al., 2025). In fact, research results of studies with participants across the United States suggest that women's fear of crime and perceived vulnerability predict their preferences for long-term mates who are aggressively dominant and physically formidable (Snyder et al., 2011). Work examining shifts in behavior across the ovulatory cycle also provide evidence in women for psychological mechanisms that protect reproductive choices by avoiding males who may present cues of sexual coercion, particularly among women who self-perceive high vulnerability to coercion when it would be most costly, such as when they are close to ovulation (McDonald et al., 2015). More work needs to be done examining these adaptively contingent preferences across cultures with varying degrees of environmental risk and individual vulnerability.

## Environmental mismatch

Considering our focus on the role of adaptive calibration of evolved psychological mechanisms that are behaviorally flexible, it's important to acknowledge that contemporary calibration processes are occurring in sociological environments which are substantially different from those encountered throughout most of human evolutionary history. Mismatch can occur when an evolved adaptation, either physical or psychological, becomes misaligned with the environment. Under such circumstances, a trait that once contributed to the fitness or reproductive success of an individual may no longer do so (Crawford and Salmon, 2002). Research on online social networks, including online dating markets and other novel social ecologies is starting to examine the role (including the possible mismatch) of these contexts in influencing the activation, calibration, and expression of a wide range of evolved psychological mechanisms. This work has the potential to help us understand the impact of social media, for example, on incels, increased rates of anxiety and depression, as well as the fertility crisis. Many years ago, Kenrick et al. (1989) demonstrated that exposure to images of highly attractive women resulted in men downgrading their assessment of their partner's attractiveness. Our mechanisms for assessing our relevant mating markets experience a mismatch in the modern world of filtered and altered images from film and television to social media none of which were a feature of our ancestral environment when no one would have seen that many highly attractive people in their lifetime (as they may now experience in 1 h online). In this sense, modern environmental inputs may cause an incorrect calibration of formerly adaptive mechanisms, in this case perceptions of a different mating pool than what is actually available to any particular individual. Similar levels of high exposure to popular attractive highly filtered and curated images of young women on social media platforms may result in anxiety among young women over their appearance and fuel not only the cosmetic industry but the cosmetic surgery industry which has seen more young patients seeking everything from laser treatments to botox and other injectables as well as more invasive procedures (Lim and Tan, 2024; Martynov and Arina, 2024). It seems likely that a better understanding of the mismatch between ancestral cues about status seeking, female hierarchies, mate preferences, and female competition could also better inform our understanding of the fertility crisis which appears to be influenced by biological, social, cultural, and economic factors (Lindahl-Jacobsen et al., 2025; Penaherrera-Aguirre et al., 2025). As well, related research on the incel phenomenon has also benefitted from looking at how the calibration of ancestral mechanisms may be affected by aspects of the modern mating market (Costello, 2025).

## Discussion

Modern incarnations of evolutionary psychological science are not, as sometimes still seen by the public or academics who may have an ideological bone to pick, focused simply on universal human psychological mechanisms, rather they focus more on the plasticity of such mechanisms, how they may be calibrated by the environment during development and across the lifespan. This approach can enhance the work being done in other fields. It has already done so to varying degrees with adjacent areas such as personality psychology, individual differences, developmental psychology, behavioral genetics and psychiatry. In the future, we would like to see more work done examining with greater detail the nature of the calibration of various mechanisms and the flexibility (or lack thereof) of the timing of calibration for shaping behavior. In addition, the question of how sensitive or susceptible individuals are to modern environmental shifts that affect the calibration of mechanisms and whether this can be buffered seems increasingly urgent with regard to questions around such topics as mental health and fertility. Some studies on disordered eating, for example, suggest that some individuals are more responsive to cues of intrasexual competition and that these perceptions may produce poorer behavioral outcomes from a health standpoint (Salmon et al., 2008; Salmon and Hehman, 2024). Other individuals are less responsive and more work on what shapes vulnerabilities and resilience against environmental mismatch will strengthen evolutionary sciences and adjacent fields. The bottom line is that, if we want to change undesirable behaviors and/or promote more desirable behaviors, we need to understand *why* and *under what circumstances* those behaviors occur. Far from just investigating human universals, this level of understanding is exactly what evolutionary-minded scientists seek to achieve.
